# Lactate metabolism and lactylation in breast cancer: mechanisms and implications

**DOI:** 10.1007/s10555-025-10264-4

**Published:** 2025-04-28

**Authors:** Yifan Qiao, Yijia Liu, Ran Ran, Yan Zhou, Jin Gong, Lijuan Liu, Yusi Zhang, Hui Wang, Yuan Fan, Yihan Fan, Gengrui Nan, Peng Zhang, Jin Yang

**Affiliations:** 1 Cancer Center, The First Affiliated Hospital of Xi’an Jiaotong University, Xi’an, China; 2 Precision Medicine Center, The First Affiliated Hospital of Xi’an Jiaotong University, Xi’an, China; 3 Department of Medical Oncology, The First Affiliated Hospital of Xi’an Jiaotong University, Xi’an, China; 4 Center for Molecular Diagnosis and Precision Medicine, The First Affiliated Hospital, Jiangxi Medical College, Nanchang University, 1519 Dongyue Dadao, Nanchang, 330209 China; 5 Jiangxi Provincial Center for Advanced Diagnostic Technology and Precision Medicine, The First Affiliated Hospital, Jiangxi Medical College, Nanchang University, 1519 Dongyue Dadao, Nanchang, 330209 China

**Keywords:** Breast cancer, Lactate, Lactylation, TME, Drugs

## Abstract

As the end-product of glycolysis, lactate serves as a regulator of protein lactylation in addition to being an energy substrate, metabolite, and signaling molecule in cancer. The reprogramming of glucose metabolism and the Warburg effect in breast cancer results in extensive lactate production and accumulation, making it likely that lactylation in tumor tissue is also abnormal. This review summarizes evidence on lactylation derived from studies of lactate metabolism and disease, highlighting the role of lactate in the tumor microenvironment of breast cancer and detailing the levels of lactylation and cancer-promoting mechanisms across various tumors. The roles of lactate and lactylation, along with potential intervention mechanisms, are presented and discussed, offering valuable insights for future research on the role of lactylation in tumors.

## Introduction

Cancer is the primary cause of illness and mortality worldwide. Breast cancer, the most common disease in women, continues to be a global public health concern. As the most common cancer worldwide, accounting for 11.7% of all cancer cases, breast cancer has surpassed lung cancer [1]. Breast cancer has a wide range of characteristics and is a very heterogeneous illness. Gene expression analysis of the disease revealed several molecular subtypes that impact prognosis. Targeted therapy for breast cancer can be categorized into four types, namely, luminal A, luminal B, HER- 2, and basal, on the basis of the hormone receptor-positive molecular subtype. Triple-negative breast cancer (TNBC), accounting for approximately 15–20% of all incidences of breast cancer, is defined as tumors that are negative for ER, PR, and HER- 2 [2, 3]. Currently, surgery, chemotherapy, radiation, endocrine therapy, and targeted therapy are the available treatment options for breast cancer. Unfortunately, side effects and drug resistance indicate that current medicines are not effective enough to achieve the desired results [4, 5]. Thus, there is a pressing need for novel therapeutic techniques to treat breast cancer.

Since lactate is thought to be a metabolic waste product of glucose metabolism under hypoxic conditions, it has long been disregarded as an end product of glycolysis. However, the identification of the Warburg effect in 1956 highlighted the importance of lactate as a byproduct and useful organic chemical generated by a high rate of glycolysis in the metabolism of cancer [6]. The Warburg effect causes the development of a cancer-promoting milieu, and lactate pathways are increasingly focused on as possible targets for tumor therapy. Higher grades of breast cancer have been shown to have elevated lactate levels, which makes understanding the role of lactate in breast cancer physiology essential [7]. The particular mechanisms and functions of the Warburg effect in carcinogenesis are not yet fully understood, despite tremendous progress over the past century. The therapeutic targeting of lactate metabolism is still in the experimental stages, with ongoing preclinical and clinical trials assessing the efficacy and safety of various inhibitors [8–11]. While targeting lactate production and transport is an exciting therapeutic approach, the nonspecific nature of these interventions may lead to significant side effects, such as disruptions in normal tissue metabolism, immune function, and the tumor microenvironment balance. Careful consideration of these risks, alongside more refined targeting strategies, is crucial to minimize toxicity and maximize therapeutic efficacy.

Posttranslational modifications (PTMs) are chemical alterations made to a protein after translation to control functions such as protein activity, localization, folding, and important interactions with other biomolecules [12, 13]. Remarkably, Zhang et al. first discovered in 2019 that the lactate-induced lactylation of histone lysine residues was implicated in the homeostatic control of M1 macrophages during bacterial infections [14]. Since then, lactate has emerged as a key player in the control of epigenetics. In addition to providing a new avenue for investigating lactate in cancer, metabolism, immunology, and other areas, the proposal of protein lactylation as a new PTM also offers a new area for the study of proteins. Unlike traditional post-translational modifications such as phosphorylation, acetylation, methylation, and ubiquitination, lactylation highlights the intersection between metabolism and epigenetics. This modification involves the direct attachment of lactate or lactyl-CoA to lysine residues on proteins, revealing how cellular metabolic states can directly influence protein function and regulate gene expression. Therefore, lactylation is not only a cellular response to hypoxic conditions but also a critical molecular link connecting metabolic changes to cellular fate decisions. Research on histone lactylation in breast cancer has been conducted to a certain extent, but there is still much room for the exploration of nonhistone lactylation, probably because the identification of modifications of nonhistone proteins has not emerged in Kla research. Despite these developments, the specific importance of Kla in tumor progression, whether as a significant driver or a minor regulator, is still being actively studied. The precise regulatory mechanisms and biological roles of lactylation are not yet known [15].

This review delves deep into lactate metabolism and the impact of histone or nonhistone lactylation on the cellular biology of breast cancer. This review helps clarify the role of lactate in the control of cell function and advances our understanding of protein lactylation. Finally, we consider the possibility of treating a variety of disorders by focusing on lactate metabolism and lactylation modification targets.

## Lactate metabolism in breast cancer

**Fig. 1:**
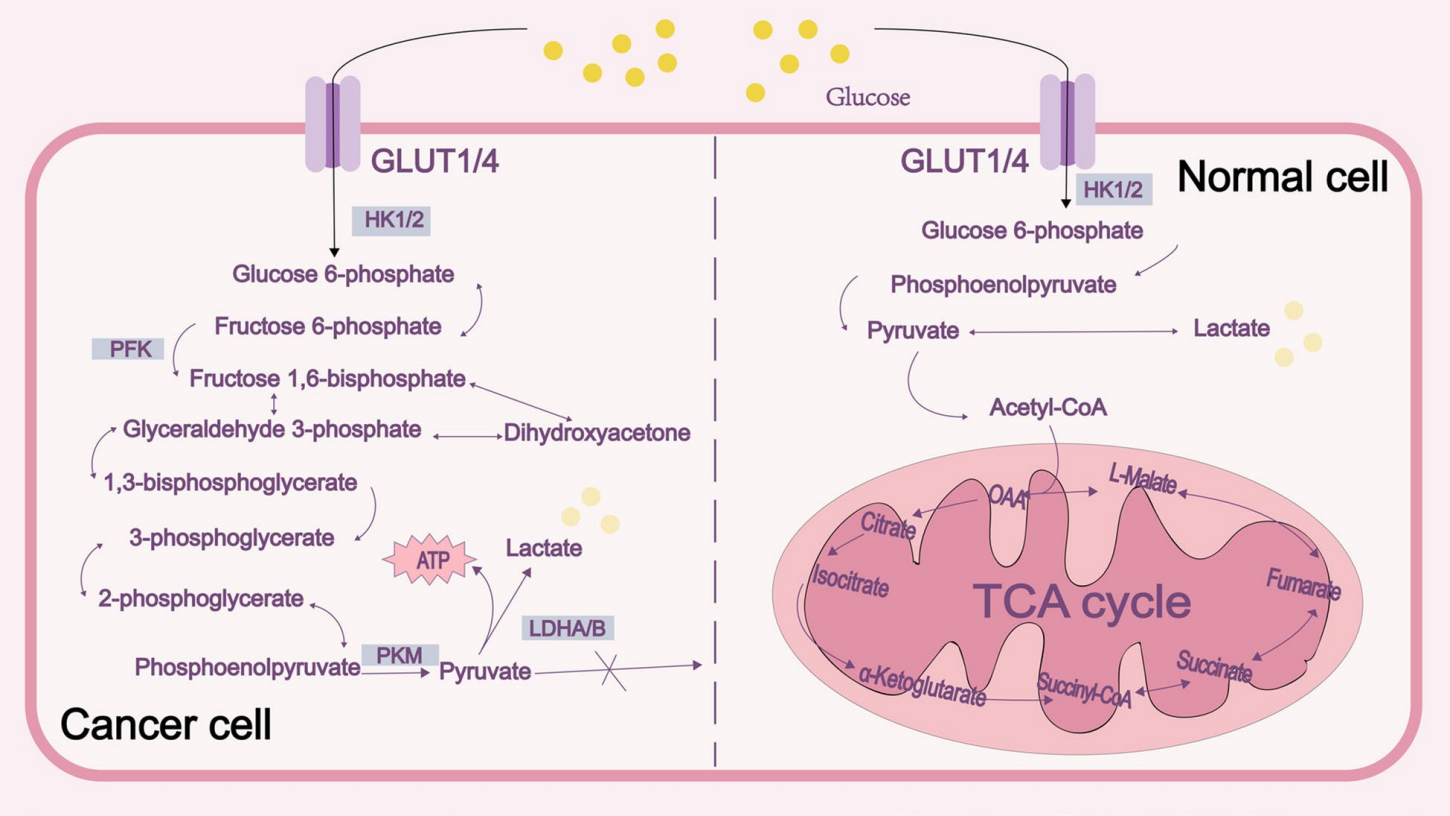
Regulation of lactate metabolism in normal and cancer cells. Glycolysis and the TCA cycle in mitochondria are the two basic processes involved in glucose metabolism. Normal cells primarily use the TCA cycle to generate energy when sufficient oxygen is present [17]. In cancer cells, glycolysis is activated in the cytoplasm when stimulated by hypoxia, malignancies, and inflammation [18]. Pyruvate is converted to lactate by LDHA, and lactate is subsequently secreted into the extracellular matrix [19]

The Warburg effect is the phenomenon by which cancer cells create ATP and lactate via glycolysis even in perfectly aerobic environments, ultimately resulting in an increase in the amount of lactate both inside and outside the cell [16]. Many processes, including the pentose phosphate system, the malate-mediated glutamine pathway, and the citrate-mediated pyruvate synthesis pathway, contribute to the generation of lactate in tumor tissue and ultimately result in the release of lactate (Fig. 1).

Lactate has a significant effect on the biological behavior of breast cancer, including angiogenesis: invasion, metastasis, growth, and metabolism. Lactate generated from tumors stimulates angiogenesis and endothelial cell activation through both HIF-dependent and HIF-independent mechanisms [20, 21]. Additionally, lactate is essential for VEGF induction, a process that is dependent on the HIF- 1 alpha signaling pathway [22]. In breast cancer, lactate binds to GPR81, promotes proliferation and stimulates angiogenesis in a PI3 K/AKT/CREB pathway-dependent manner [23, 24]. Lactate has also been shown to promote tumor invasion and metastasis. Lactate acts as a metabolic coupling link between CAFs and CTCs through MCT4/MCT1, activating the TGFb1/P38 MAPK/MMP2/9 signaling axis to increase the mitochondrial activity of CTCs and promote TNBC metastasis [25]. By increasing the oxidative phosphorylation of breast cancer cells, lactate may promote distant metastasis [26]. Aerobic glycolysis speeds up glucose metabolism, but tumor cells use increased glycolysis to meet their energy needs for cancer cell growth and proliferation [18]. Moreover, lactate can regulate important energy metabolism enzymes, such as pyruvate kinase 2 (PKM2), to support the growth and metabolism of cancer cells [27–29]. Therefore, lactate metabolism in breast cancer plays a critical role in tumor progression and behavior, warranting increased attention in both research and clinical treatment.

### Glycolytic reprogramming increases the production of lactate in breast cancer

Breast cancer cells are characterized by high glucose uptake, high energy demand, and high glycolysis rates. One of the main hallmarks of cancer is metabolic reprogramming, which involves changes in the properties of metabolic enzymes, upstream regulatory molecules, and downstream metabolic products, i.e., metabolites [30–32]. The following related molecules are implicated in glycolytic reprogramming: glucose transporters (GLUTs), lactate dehydrogenase (LDH), phosphofructokinase (PFK), and pyruvate kinase (PK) [33–35].

Proteins in the GLUT family help carry glucose into cells, where it is oxidized to produce ATP in the mitochondria. A low survival rate of patients with breast cancer is associated with high Glut- 1 expression [36]. In addition, breast cancer patients with lymph node metastasis and high Glut- 1 expression have poor overall survival, a high histological grade and a large tumor size [37]. Research has shown that even under hypoxic conditions, the WZB117-induced blockage of the glucose transporter GLUT- 1 causes metabolic abnormalities in HER- 2-overexpressing breast cancer cells [38]. The key enzyme involved in the conversion of pyruvate to lactate after aerobic glycolysis is LDH. Research has revealed that LDHA inhibits the invasion of antitumor immune cells and promotes the maintenance of breast cancer stemness [39]. Through the increase in HSF1, ErbB2 overexpression increases LDHA, which in turn leads to an increase in glycolysis in human breast cancer cells [40]. The buildup of lactate caused by LDHA promotes angiogenesis and extracellular matrix degradation. Breast cancer malignancy can be categorized by the level of LDHA expression [41]. Research has shown that the phosphorylation of LDHA at tyrosine- 10 (Y10) increases its catalytic activity, increasing the capacity of breast cancer cells for metastasis and resistance to anoikis. Furthermore, in tissue samples from breast cancer patients, phosphorylated LDHA is strongly linked with the development of metastases [42]. The second irreversible and regulating step in glycolysis is the conversion of fructose- 6-P into fructose- 1,6-bis-P via phosphofructokinase. G. Wang et al. reported that as the clinical stage of breast cancer progresses, the efficiency of glycolysis and the expression of PFK- 1 increase [43]. Preliminary research has shown that the phosphorylation of PFKFB3 at Ser478 stimulates the proliferation and glycolysis of breast cancer cells. Additionally, paclitaxel resistance in breast cancer is conferred by PFKFB3 stability both *in vitro* and *in vivo* [44]. Recent research has demonstrated that PFKFB3 controls the cell cycle of breast cancer cells by downregulating p27 expression via AKT phosphorylation [45, 46]. The overexpression of PFKFB3 affects several cellular processes, such as angiogenesis, stemness, metastasis, treatment resistance, and survival, in cancer cells [46]. In glycolysis, pyruvate kinase (PK) is an essential enzyme capable of converting phosphoenolpyruvate into pyruvate, which has four isoforms, including the liver isoform (PKL), the erythrocyte isoform (PKR), and two muscle isoforms (PKM1 and PKM2). According to recent research, breast tumor cells expressing high levels of PKM2 are less sensitive to the anticancer medications 5-fluorouracil and epirubicin [47]. Furthermore, TNBC cells are more sensitive to doxorubicin therapy after PKM2 suppression [48].

### Monocarboxylic acid transporters (MCTs)—lactate transmembrane transporters in breast cancer

There are three known methods by which lactate traverses the cell membrane: transporters, anion exchange, and free diffusion. Specific transporters, including MCTs, play a significant role in the lactate shuttle. MCTs play two roles in cancer metabolism as proton symporters: they regulate pH and extrude (and invade) lactate [49]. MCTs are members of the SLC16 gene family, which consists of 14 members. Tumor cells that are close to blood arteries, where oxygen is more readily available, can absorb lactate from the matrix via MCT1 (SLC16 A1) and oxidize it to produce ATP [50]. MCT4 (SLC16 A3) transports the lactate generated in tumor cells in hypoxic areas, far from blood arteries, out of the cells and into the extracellular matrix. MCT- 1 serves as a negative prognostic indicator for aggressive breast cancer, as it facilitates IL- 6/IL- 6R/gp130 signaling-mediated epithelial‒mesenchymal transition (EMT) and the progression of TNBC [51]. The systemic targeting of MCT- 1/IL- 6/IL- 6R/CXCL7/PD-L1 linkages increases immune surveillance, hence inhibiting the aggressiveness of TNBC [52]. According to reports, MCT4 is overexpressed in breast cancer, especially in the basal-like molecular subtype [53]. This evidence highlights the important roles that MCTs and lactate play in breast cancer.

In conclusion, MCTs, especially MCT1 and MCT4, play critical roles in lactate transport and contribute significantly to the metabolic adaptation of breast cancer cells. Furthermore, targeting MCTs and their associated signaling pathways may offer promising therapeutic strategies for controlling breast cancer aggressiveness and enhancing immune response.

### Lactate affects the breast cancer tumor microenvironment (TME)

The TME is a multifaceted ecosystem consisting of diverse cellular and noncellular elements, such as tumor cells, immune cells, blood vessels, stromal cells, and molecular signaling networks [54–56]. High levels of aerobic glycolysis and glutamine glycolysis in tumors lead to the significant release of lactate and H + into the extracellular space [57], causing the accumulation of the end product lactate in the TME, where the concentration can reach 40 mM [58], such as in breast cancer. Under normal conditions, the lactate concentration in blood and healthy tissues ranges from 1.5 to 3 mM [57].

**Fig. 2:**
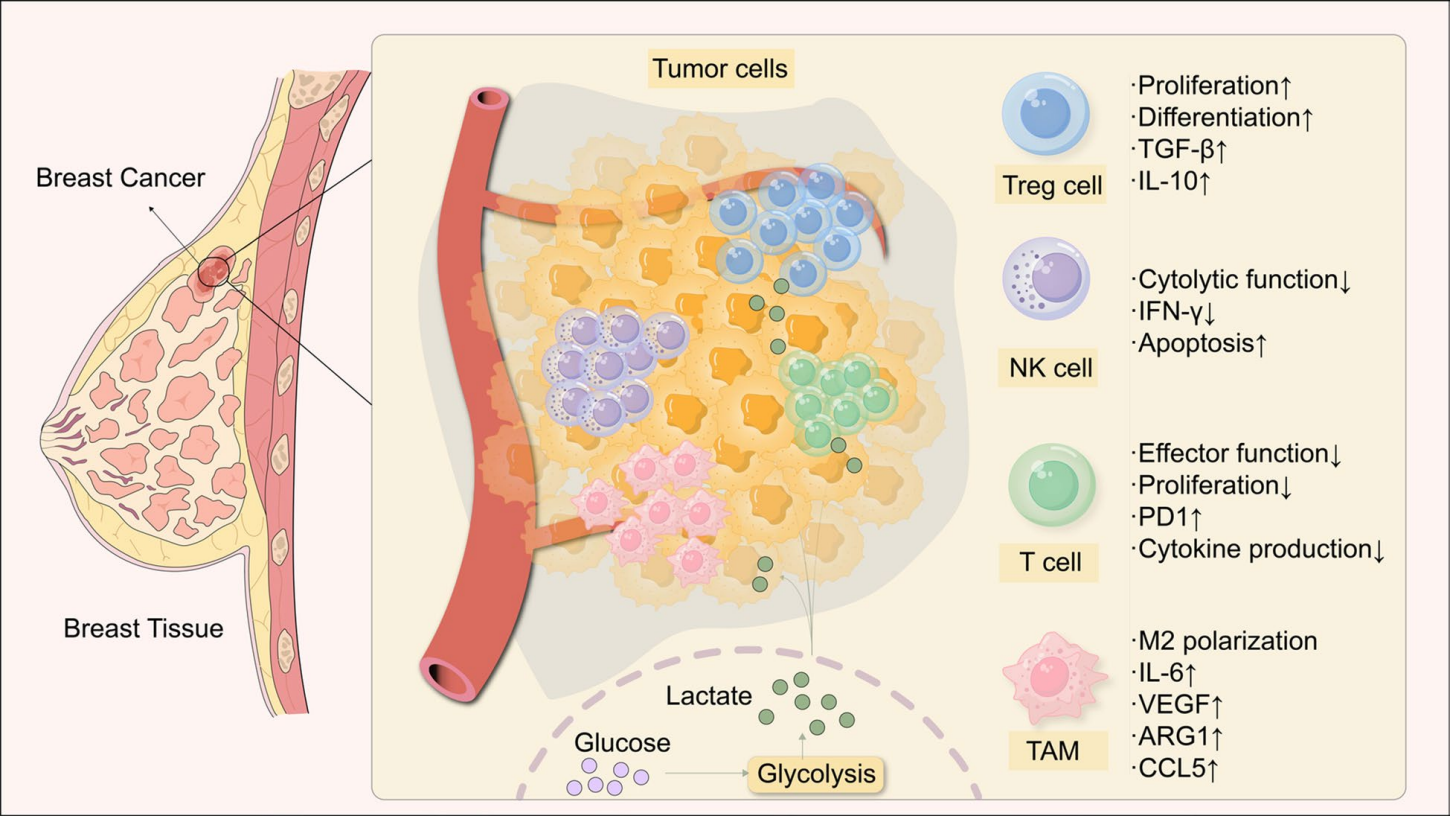
Lactate affects the immune microenvironment in breast cancer. The tumor microenvironment is composed of cancer cells, tumor-associated immune cells, and tumor-associated fibroblasts, some of which have been shown to be lactate regulated. Lactate can aid Tregs in suppressing the growth and activity of other immune cells, such as by reducing the potency of T and NK cell cytotoxicity [66]. Moreover, lactate causes TAMs to differentiate into an M2-like phenotype, which is associated with the immune suppression of tumor invasion and cancer metastasis, but it also inhibits TAMs from M1 polarization, which mediates antitumor inflammation and the immune response [60]. Additionally, medium acidification decreases the pH of immune cells and suppresses the functions of T and NK cells, among other immune cells; however, it is yet unclear whether lactylation affects these immune cells. Furthermore, many cells in the tumor microenvironment that are directly linked to the growth of tumors may also absorb lactate

Lactate serves as both an energy source and a signaling molecule for tumor growth at various levels, driving tumor cell proliferation [21]. Lactate stimulates breast cancer cell adhesion, migration, invasion and Akt activity via GPR81 [24]. The lactate-rich tumor environment mediates immunosuppression via tumor-associated macrophages, natural killer cells, T regulatory cells, and T lymphocytes. TAMs, or tumor-associated macrophages, are prevalent in tumors and polarize between proinflammatory “M1” and anti-inflammatory “M2” subtypes in response to external stimuli [59]. Lactate activates the ERK/STAT3 signaling cascade, which promotes angiogenesis, migration, and cell proliferation in breast cancer by driving polarized macrophages to the M2 type [23] (Fig. 2). By attaching itself to the lactate-sensitive receptor GPR132, tumor-derived lactate can cause TAM polarization toward the M2 immunosuppressive phenotype [60, 61]. TAM-specific extracellular signal-regulated kinase (ERK)/STAT3 activation, increased vascular endothelial growth factor (VEGF) and arginase- 1 (ARG1) production, and HIF- 1a stabilization have all been implicated in lactate-induced TAM polarization and its protumorigenic effects in breast cancer [23, 60, 62]. It has been reported that lactate causes human macrophages to become TAM-like and that CCL5 is secreted in response to Notch signaling in macrophages. Additionally, CCL5 promotes aerobic glycolysis in breast cancer cells, induces EMT in cancer cells, and increases cell migration, all of which indicate the presence of a positive metabolic feedback loop in coculture systems [63]. LDHB production in tumors inhibits the secretion of lactate, which in turn activates NK cells to prevent the growth of malignancies [64]. Tumor-associated macrophages and T regulatory cells are favored by hypoxia and an acidic TME, but antitumor immune responses are inhibited. With research identifying a group of lactate-related genes (LRGs), lactate scores have recently shown promise as an independent predictive indicator for BC. The prognosis of BC patients and TME immune cell infiltration can both be assessed using the lactate score. In particular, a low lactate score is linked to immunological activation, which includes an inflammatory TME and increased CD8 + T cell infiltration [65]. One essential element of the TME is stromal fibroblasts, also known as CAFs [57]. Recent research has shown that lactate transfer is how CAFs power BC cells. The lactate generated by CAFs ultimately promotes the invasion of BC cells by activating the TGFβ1/p38 MAPK/MMP2/9 signaling pathway and enhancing mitochondrial OXPHOS [25] (Fig. 2).

## Lactylation: a new protein posttranslational modification

### Histone lactylation affects the transcription of crucial genes

As high-throughput sequencing technologies have advanced, an increasing number of new epigenetic codes, such as lactylation, are being identified. Histone lactylation has been shown to modulate gene transcription via epigenetic processes. In 2019, Zhang et al. first identified a new form of core histone lysine lactylation (Kla) in human MCF- 7 cells [14]. They discovered that arginase 1 (Arg1) is a Kla-modified gene, which means that the expression of the M2-like gene Arg1 is positively associated with histone Kla levels [14]. A significant milestone in the investigation into the precise function of histone lactylation in various solid tumors was the discovery of its connection to melanoma [67]. Transcriptional regulation is fundamentally dependent on histone modifications, and Kla induces structural alterations in chromatin. These modifications impact the interaction of transcription complexes with gene promoters as well as the affinity of histones for DNA. The impact of histone lactylation on gene expression remains ambiguous, as the majority of research has concentrated solely on particular gene sets. Histone lactylation may be context-dependent in its control of gene transcription, which emphasizes the necessity of thorough investigations that integrate RNA sequencing and histone lactylation CUT-TAG [68]. In breast cancer cells, elevated intracellular lactate levels cause H3 K18 la enrichment in the − 70 to + 3 promoter area, which increases c-Myc expression; this, in turn, controls SRSF10 to affect alternative splicing of MDM4 and Bcl-x [69]. According to another investigation, KCNK1 activates LDHA and upregulates H3 K18 lactylation to stimulate the growth and metastasis of breast cancer cells [70]. In addition to the widely studied H3 K18 la, histone H4 K12 lactylation accelerates the development of TNBC by downregulating SLFN5 expression [71]. Our understanding of epigenetics has been significantly expanded by histone Kla.

### Nonhistone lactylation influences protein functions

Some of the lactate that cells create during the dynamic metabolic homeostasis of tissues is used for metabolism, and the remaining portion is absorbed to take part in nonhistone lactylation and epigenetic modifications. Research has shown that lactylation is widespread [72, 73]; hence, investigating how lactylation affects nonhistone proteins with a variety of functions is crucial. Research on the lactylation of nonhistone proteins has demonstrated that this alteration can either increase or decrease protein natural activity. However, it is uncertain whether lactylation can bestow completely new activities. First, 2375 Kla sites were found in a thorough examination of gastric cancer (GC) cells, and the related proteins were significantly enriched in spliceosome function [74]. This was the first lactylome analysis in tumors and advanced the understanding of lactylation function and control in both healthy and pathological settings and broadened the database of human lactylation. Zhou et al. reported that lactylation inhibits p53 liquid‒liquid phase separation, binding to DNA, and target gene induction, contributing to carcinogenesis, including that of breast cancer. They also identified two lactylation residues, K120 and K139, within the p53 DNA-binding domain as legitimate targets of AARS1 [75]. Platinum-based chemotherapy remains the mainstay of treatment for triple-negative breast cancer (TNBC). But after stopping the drug, TNBC cells usually relapse and become resistant to platinum. According to Mei et al., methyltransferase-like 3 (METTL3) is delactylated by histone deacetylase 2 (HDAC2), which facilitates METTL3’s interaction with Wilms’ tumor- 1-associated protein and raises m6 A of transcript-associated DNA damage repair. The biological role of lactylated METTL3 in tumor cells is clarified by this study, which also emphasizes how it negatively regulates cisplatin resistance [76]. Modifications, stability, or expression variations in proteins are typically associated with an organism’s functions. Few studies have investigated nonhistone lactylation in breast cancer; however, nonhistone lactylation has emerged as a key regulatory mechanism in a variety of biological processes. Therefore, it needs to receive more attention.

### Key enzymes involved in lactylation

**Fig. 3:**
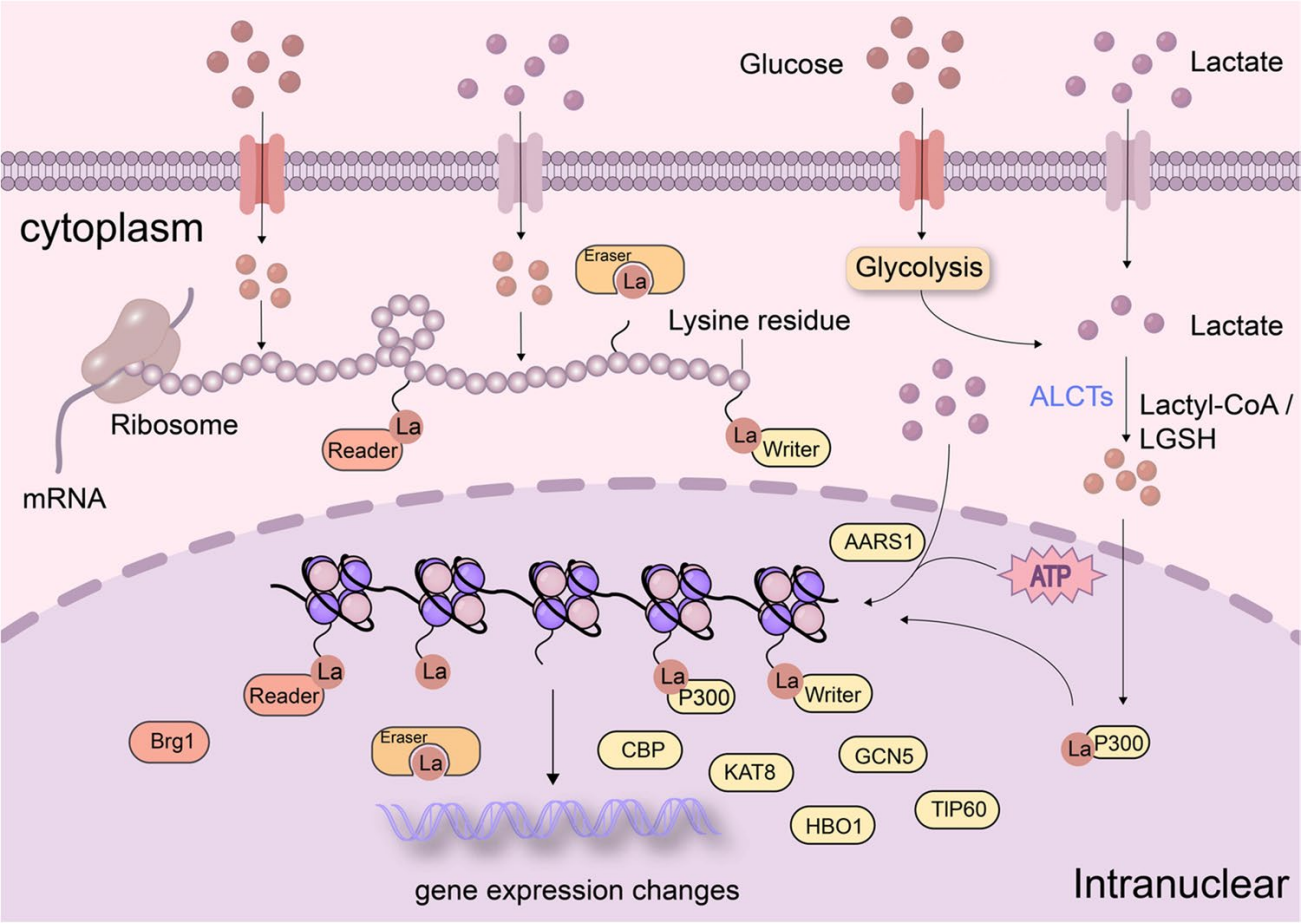
The process of lactylation. Glycolytic cells strongly consume glucose and break down glucose in the cytoplasm via glycolysis, during which LDHA converts pyruvate to lactate, which is subsequently involved in the lactylation of histones or nonhistones. Lactate can be directly involved as a substrate in the process of lactylation [75, 85]. Lactyl-CoA can be produced by lactate, and once it is present, the lactyl group is transferred to lysine via a process known as “writing,” which can affect downstream signaling pathways or the expression of certain genes via the lactylation of histones or nonhistones [77–80]

The presence of Kla is contingent upon essential enzymes. First, “writers” are primarily classified into two principal categories according to their substrate. In most of the reported lactylation-modified proteins, the target protein is attached to the lactyl moieties of lactyl-CoA from L-lactate via the ε-amino group of lysines. Lysine histone acetyltransferases (HATs) facilitate the transfer of lactyl groups from lactyl-CoA. P300/CBP, GCN5 and KAT8 have been shown to perform writer roles in the process of lactylation [77–80]. HBO1 has also been identified as a lactyltransferase that catalyzes histone H3 K9 la [81]. TIP60 has been shown to possibly be the lysine lactosyltransferase responsible for NBS1 emulsification [82]. Using lactate from glycolysis, nuclear GTPSCS/p300 produces lactyl-CoA for *in situ* histone H3 K18 la, which controls GDF15 expression to promote glioma growth and radioresistance [83]. According to Zhu et al., ACSS2 is a genuine lactyl-CoA synthetase that transforms lactate into lactyl-CoA. The ACSS2/KAT2 A complex is formed in response to EGFR activation by ERK phosphorylation-mediated nuclear translocation of ACSS2, which acts as a lactyltransferase to enhance histone lactylation, gene expression, tumor development, and immune evasion [84]. However, the concentration of lactyl-coenzyme A is approximately 1000 times less than that of acetyl-CoA. The enzyme alanyl-tRNA synthetase 1 (AARS1) has been found to double as a genuine lactyl-transferase, catalyzing protein lactylation directly with the help of ATP and lactate [75, 85]. Mitochondrial alanyl-tRNA synthetase (AARS2) acts as a protein lysine lactyltransferase [86] (Fig. 3). The “erasers”, a set of highly effective enzymes that eliminate lactyl groups, are known as histone lysine deacetylases (HDACs), which are further subdivided into the SIRT2 family of NAD + -dependent HDACs and the classical HDAC family [87]. Furthermore, HDAC1 - 3 and SIRT1 - 3 are effective in vitro erasers of Kla [88]. Finally, this change in Kla is particularly recognized by effector proteins known as “readers,” which then alter downstream signaling pathways and initiate a variety of cellular processes. Hu identified the binding of Brg1 to H3 K18 la, highlighting its function as a reader of histone lactylation [89]. Additionally, the transformation of lactate by ALCTs into lactyl-CoA is a crucial stage in the Kla process. Zhang et al. screened, cloned, produced, purified, and characterized five possible lactyl-CoA-converting enzymes and examined the stability of the three ALCTs with the highest specific enzymatic activity [90].

### Nonenzymatic lactylation of lysine

The concentration of lactyl-CoA is 1/350 to 1/20 that of acetyl-CoA, propionyl-CoA, or other typical acyl-CoAs in HepG2 cells 91]. Given that lactyl-CoA and lactate deposition occur at relatively low levels, the enzymatic regulation of lactylation is extremely unlikely in most circumstances, and additional lactylation-related mechanisms may exist. In contrast to the mechanism discovered by Zhao [14], Dominique et al. discovered a novel nonenzymatic lactate pathway with lactate glutathione (LGSH) as the substrate instead of lactate coenzyme A, as in conventional studies [92].

### The multifaceted roles of protein lactylation in cancer

Although lactylation was first identified in studies on immunity and inflammation, the findings suggest that it may also play a role in the development of cancer. Lactate, a primary metabolite generated by the Warburg effect, has garnered increased attention since Otto Warburg’s 1956 discovery [6]. With advancements in research, the pivotal role of lactate in the progression of cancer has been increasingly confirmed, moving from metabolic waste to a “general fuel” and “information transmitter” and finally to an important substrate for metabolic reprogramming [24, 93]. High amounts of lactate in the microenvironment are a significant underlying condition for lactylation [94], meaning that the whole TME, including tumor parenchymal cells, stromal cells, and even immune cells, may have significantly increased lactylation modification levels. The process by which protein lactylation controls the growth of tumors is now being progressively elucidated by an increasing number of studies. Lactylation alters a variety of intercellular connections and functions in this setting, opening new possibilities for better understanding and treating cancer.

#### Direct effects of lactylation on tumor cells

Lactylation is a distinctive form of lactate modification that may be essential in the progression of cancer. Depending on the proteins that are largely impacted by lactylation, the possible effects of lactylation can be categorized into histone-mediated pathways and nonhistone-mediated pathways. Differences in histone lactylation levels among various cancer types can result in unique gene expression profiles, affecting cellular development and fate. The lactylation of nonhistone proteins may result in diverse functions under distinct biological situations.

The lactylation-induced epigenetic modification of gene expression that modifies tumor characteristics is known as the histone-mediated pathway. In ocular melanoma, augmented histone lactylation enhances the transcription of YTH domain family protein 2 (YTHDF2), facilitating the degradation of m6 A-modified PER1 and TP53 mRNAs and hence expediting tumorigenesis [67]. Wei et al. reported that H3 K18 la triggered TTK and BUB1B transcription in PDAC cells to promote the cell cycle and accelerate carcinogenesis [95]. In bladder cancer, H3 K18 lactylation increases the expression of the oncogene lipocalin- 2 (LCN2), facilitating tumorigenesis. Nonetheless, the precise processes by which LCN2 facilitates bladder cancer progression have yet to be completely clarified [96]. By promoting platelet-derived growth factor β (PDGFRβ) transcription through H3 K18 la, the inactivation of VHL, a key prognostic factor in ccRCC, creates a positive feedback loop that accelerates the development of ccRCC [97]. Histone H3 K9 la stimulates LAMC2 transcription, which facilitates the invasion and growth of esophageal cancer cells [98]. In endometrial carcinoma, histone lactylation facilitates USP39 expression to target the PI3 K/AKT/HIF- 1 t signaling pathway, which advances cancer treatment [99]. However, another study revealed that lactate-induced H4 K12 lactylation in TNBC cells preferentially inhibits SLFN5 expression, which in turn contributes to TNBC malignancy [71]. The role of histone lactylation in gene expression remains ambiguous, as it is uncertain whether it universally inhibits or enhances this process.

The term “nonhistone-mediated pathway” describes how lactylation affects overall protein structure, function, and location. As demonstrated by Liang et al., nucleolar and spindle-associated protein 1 (NUSAP1) Kla modulates the expression of LDHA in pancreatic cancer; this results in the formation of an NUSAP1 Kla-LDHA-glycolysis-lactate feed-forward loop, serving as a possible mechanism underlying the metabolic propensity of glycolysis in pancreatic cancer [100]. Tumor angiogenesis involves a network of blood vessels that provide tumors with a favorable milieu for either local or distant metastasis and is the primary cause of most cancer-associated death events [101]. The proangiogenic role of KIAA1199 in prostate cancer has been suggested to be triggered by the MCT1-mediated elevation of lactate, which is thought to increase HIF1a lactylation and stimulate hyaluronan (HA)-binding protein KIAA1199 signaling. These findings may provide a viable target for the treatment of antiangiogenic tumors [102]. Hypoxia-induced glycolysis has been shown to increase β-catenin lactylation, stability, and levels, all of which promote the malignant growth of colon rectal cancer (CRC) cells. Moreover, hypoxia increases the lactylation and protein level of SHMT2, which increases MTHFD1L expression and accelerates the malignant development of EC cells [103]. Glycolysis promotes P300-catalyzed nucleolin lactylation, which triggers ERK signaling and upregulates MAP kinase-activating death domain protein (MADD) expression through accurate mRNA splicing to cause intrahepatic cholangiocarcinoma (iCCA) formation [104]. AARS1 senses intracellular lactate and translocates into the nucleus to lactylate and initiate the YAP-TEAD complex [85]. Moreover, numerous AARS1 targets, including p53, whose DNA binding domain contains lactylated lysines 120 and 139, have been identified via proteomics investigations. AARS1-induced p53 lactylation prevents p53 liquid–liquid phase separation, DNA binding, and transcription initiation, therefore diminishing the tumor-suppressive functions of p53 *in vitro* and *in vivo* [75] (see Table 1).

**Table 1:** Role of lactylation in various cancer types and sites

Cancer		Lactyltransferase (writers)	Delactylase (erasers)	Lactylation site	Function and mechanisms	References
Breast cancer				H3 K18 la	When KCNK1 binds to and activates LDHA, it increases histone lysine lactylation, increases the expression of downstream targets, and speeds up cellular glycolysis and lactate generation	[70]
				H3 K18 la	Histone lactylation-dependent c-Myc induction is facilitated by intracellular lactate	[69]
Colorectal cancer (CRC)				H3 K18 la	The therapeutic effectiveness of bevacizumab in colorectal cancer is enhanced by preventing histone lactylation	[105]
				H3 K18 la	The transcription of the RARg gene in macrophages is prevented	[106]
				H3 K18 la	Mettl3 expression in tumor-infiltrating myeloid cells is elevated by H3 K18 lactylation	[107]
				H3 K18 la	GPR37 enhances H3 K18 lactylation to boost neutrophil recruitment	[108]
				β-catenin	Glycolysis triggered by hypoxia facilitates the lactylation of β-catenin, increasing the protein stability and expression of β-catenin and thus exacerbating the malignant characteristics of CRC cells	[109]
				H4 K8 la	LPS-H4 K8 la-LINC00152 induces the migration and invasion of tumors	[110]
		KAT8		eEF1 A2	The lactylation of eEF1 A2 K408 leads to increased protein synthesis and translation elongation, both of which aid in tumor development	[80]
Gastric cancer				H3 K18 la	An increase in VCAM1 transcriptional activity leads to GC cell migration and proliferation as well as a poor prognosis for GC patients	[111]
			SIRT2	METTL16 K229	The lactylation of METTL16 at K229 increases FDX1 protein expression via the m6 A modification of FDX1 mRNA, hence activating cuproptosis under copper stress	[112]
		AARS1	SIRT1	YAP-TEAD	AARS1 stimulates GC growth in response to the lactylation of YAP-TEAD	[85]
Hepatocellular carcinoma				H3 K9 la, H3 K56 la	Histone lactylation can promote tumor growth, metastasis, and malignant characteristics by increasing the expression of ESM1 in HCC	[113]
Intrahepatic cholangiocarcinoma		P300		Nucleolin	The lactylation of nucleolin triggers ERK signaling to enhance iCCA development and upregulates MAP kinase-activating death domain protein (MADD) expression through accurate mRNA splicing	[104]
Pancreatic ductal adenocarcinoma		P300	HDAC2	H3 K18 la	H3 K18 la promotes TTK and BUB1B transcription in PDAC	[95]
Lung cancer				H3 K18 la	H3 K18 la activates the POM121/MYC/PD-L1 pathway to enhance the immunological escape of NSCLC cells	[114]
				H4 K8 la, H4 K16 la,H3 K14 la	Histone lactylation modulates the transcriptional activity of Sp1 and controls telomerase activity	[115]
				IGF1R	Increased IGF1R lactylation is linked to the IGF1R-mediated aggressive tendencies of LC cells	[116]
				SOX9	By inducing SOX9 lactylation, hypoxia facilitates the stemness, motility, and invasion of NSCLC cells	[117]
Non-small cell lung cancer				H3 K18 la	Lactate affects cellular metabolism, at least in part through histone lactylation-mediated gene expression	[118]
Endometrial cancer				H3 K18 la	Histone lactylation affects USP39 expression, and USP39 interacts with PGK1 to activate the PI3 K/AKT/HIF- 1α signaling pathway, which in turn increases lactate production	[99]
Ovarian cancer				H3 K18 la	The carcinogenesis of ovarian cancer is induced by the production of CCL18 in macrophages through H3 K18 lactylation	[119]
Glioblastoma				H3 K9 la	Recurrent GBM tissues and TMZ-resistant cells show elevated lactylation	[120]
				XRCC1	Lactylated XRCC1 has a higher affinity for importin a; it can translocate to the nucleus and improve DNA repair	[121]
Bladder cancer				H3 K18 la	The circXRN2-Hippo pathway regulatory axis slows tumor growth by reducing H3 K18 lactylation and LCN2 expression in human bladder cancer	[96]
				ZEB1	ZEB1 is inhibited by PFK- 1 to decrease lactylation, which inhibits BC	[122]
Esophageal cancer				SHMT2	The malignant growth of EC cells is accelerated by the hypoxia-induced the lactylation of the SHMT2 protein and an increase in its protein level, leading to an increase in MTHFD1L expression	[103]
				Axin1	Axin1 lactylation promotes Axin1 ubiquitination, facilitating protein degradation and anti-glycolytic action	[123]
Cervical cancer				G6PD	The carcinogenic effects of HPV are lessened by increasing G6PD lactylation in HPV-infected cells or by inhibiting G6PD enzymatic activity with 6 An	[124]
				DCBLD1	DCBLD1 expression is stabilized by lactate-induced DCBLD1 lactylation	[125]
Ocular melanoma				H3 K18 la	Histone lactylation at high levels enhances the expression of YTHDF2, which in turn binds to the m6 A sites of PER1 and TP53 mRNAs to degrade the RNA	[67]
Clear cell renal cell carcinoma				H3 K18 la	Histone lactylation induced by inactive VHL promotes ccRCC progression by stimulating the transcription of platelet-derived growth factor receptor β (PDGFRβ)	[97]
Prostate cancer				H3 K18 la	Lactate transported into PCa cells via MCT1 stabilizes HIF1α under normoxia by lactylation	[102]
Pancancer	Pancreatic adenocarcinoma	EP300	SIRT1	NMNAT1	To support nuclear translocation and preserve enzymatic activity, L-lactate increases NMNAT1 lactylation	[126]
	Cervix cancer, hepatocellular carcinoma, glioblastoma, breast cancer, lung cancer, and esophageal cancer	HBO1		H3 K9 la	Tumor initiation gene transcription may be triggered by HBO1-mediated H3 K9 la	[81]
	Lung cancer, melanoma (type of skin cancer), and colorectal cancer			H3 K18 la	Mettl3 expression in tumor-infiltrating myeloid cells increases via H3 K18 lactylation	[107]
	Gastric cancer, lung cancer, colorectal cancer, cervical carcinoma, and osteosarcoma	TIP60	HDAC3	NBS1 K388	The inhibition of NBS1 K388 lactylation decreases DNA repair efficacy and reverses resistance to chemotherapy	[82]
	Cervical carcinoma, osteosarcoma, non-small cell lung cancer, and colorectal cancer	AARS1		P53	Because lactylation prevents p53 LLPS from binding to DNA and inducing its target genes, it plays a role in the development of tumors	[75]
	Osteosarcoma, breast cancer, and colorectal cancer	CBP		MRE11 K673	The lactylation of MRE11 enhances its affinity for DNA, hence promoting DNA end resection and homologous recombination	[127]

#### Lactylation of immune cell and tumor progression in TME

TME alterations have a significant impact on carcinogenesis. One of the most common metabolites in the TME is lactate. TAMs have a greater degree of histone lysine Kla than do other cells. These findings suggest that Kla significantly regulates the TME, potentially opening new avenues for targeted, antiangiogenic, and tumor immunotherapy treatments. In the TME, immune cells have major anticancer effects. They have the ability to monitor the levels of extracellular lactate and deliver intracellular signals that regulate how they function [128]. Lactylation changes impact the activity of different immune cells by changing their metabolic state and signaling pathways.

Macrophages, which exhibit M1 and M2 phenotypes, are among the most significant types of innate immune cells in the human body. The M2 phenotype, which inhibits the immune system and encourages tumor growth and metastasis, is present in 70% of TAMs [129]. According to Zhao et al., when M1 macrophages are activated, glycolysis is triggered, which results in the production of a significant amount of lactate and an increase in H3 K18 la. The expression of proteins linked to the M2 phenotype can be upregulated by histone Kla, suggesting that histone Kla facilitates the transition of TAMs from a proinflammatory, anticancer M1 phenotype to an anti-inflammatory, procancer M2 phenotype [14]. Zhao et al. reported histone lactylation modifications not only in human breast cancer cells but also in macrophages isolated from mouse melanoma and lung tumors; importantly, they reported a positive correlation between the level of histone lactylation and the propensity of M2 macrophages to cause cancer [14]. These findings imply that tumor development and progression may be facilitated by increased histone lactylation in M2 macrophages. Patnaik et al. reported that decreasing phosphatidylinositol- 3 kinase (PI3 K) led to a decrease in lactate production in tumor cells, which inhibited the histone Kla of TAMs and increased immune efficacy [130]. Another investigation revealed that the Kla of PKM2 inhibits glycolysis and promotes macrophage polarization from the M1 to M2 phenotype [131]; additionally, it contributes to the carcinogenesis of ovarian cancer by inducing the production of CCL18 in macrophages through H3 K18 lactylation [119]. Tumor-derived lactate stimulates H3 K18 lactylation in macrophages in colorectal cancer, which inhibits the transcription of the RARcta Biochimincreases interleukin- 6 (IL- 6) levels in the TME. In CRC cells, this then triggers the STAT3 signaling pathway, which promotes the transcription of the c-Myc gene and changes macrophages into protumor M2-like cells [106]. Histone lactylation caused by PERK-driven glucose metabolism enhances MDM immunosuppressive activity. Tumor-specific CTLs are more sensitive to AICD by histone lactylation-activated circATXN7, demonstrating the significance of histone lactylation in controlling T cell-mediated tumor immunological escape [132]. H3 K18 la activates the POM121/MYC/PD-L1 pathway, which enhances the immune escape of NSCLC cells. Thus, intratumoral CD8 + T cell activity was increased, and high antitumor efficacy was demonstrated by combination therapy involving a glycolysis inhibitor and an anti-PD- 1 antibody [114].

Kla also affects Th17 cells. Lactate treatment of Th17 cells upregulated Foxp3 expression, which is associated with H3 K18 la [133]. Additionally, by directly affecting the expression of Th17-associated genes such as runt-related transcription factor (Runx1), Toll-like receptor 4 (Tlr4), interleukin- 2 (IL- 2), and interleukin- 4 (IL- 4), Ikaros family zinc finger 1 (IKZF1) K164 lactylation can improve Th17 differentiation [134]. Kla is a key player in controlling the development of Th17 cells. In summary, the identification of Kla represents a novel approach to tumor immunotherapy.

#### Lactylation in breast cancer: promoting tumor development and progression

In human MCF- 7 cells, 26 histone Kla sites have been identified by MS analysis [14]. In MCF- 7 cells, the rates of U- 13 C6-glucose incorporation by histones Kla and Kac vary. Additionally, intracellular lactate levels and histone lactylation are affected by influencing the activity of enzymes involved in glycolysis and mitochondrial metabolic pathways or by modeling hypoxia. In Pandkar’s study, intracellular lactate was shown to stimulate histone lactylation-dependent c-Myc activation utilizing mutants of breast cancer cell lines with a lactate-deficient metabolome. According to their findings, serine/arginine splicing factor 10 (SRSF10) expression is upregulated by c-Myc to promote alternative splicing in breast cancer cells. Therefore, chemotherapeutic therapies that reduce the glycolytic rate may limit the growth of breast cancer by blocking the c-Myc-SRSF10 axis [69]. Surprisingly, further research revealed that KCNK1 expression is considerably upregulated in human breast cancer and is associated with a poor prognosis for breast cancer patients. KCNK1 is bound to and activates lactate dehydrogenase A (LDHA), which enhances histone lysine lactylation to induce the expression of several downstream genes as well as LDHA itself; this increases glycolysis and lactate generation in breast cancer cells. Notably, elevated LDHA expression acts as a vicious positive feedback loop to decrease tumor cell adhesion and stiffness, ultimately leading to breast cancer spread, invasion, and proliferation [70] (see Table 1). Another study revealed that lactate-induced H4 K12 lactylation in TNBC cells preferentially inhibits SLFN5 expression, which in turn contributes to TNBC malignancy. These discoveries provide fresh perspectives on the processes that underlie the genesis and progression of breast cancer and could help create efficient therapeutic approaches that target important enzymes that control tumor metabolism or mediate the transcriptional regulation of target genes [71]. Regarding differentially expressed lactylation sites in triple-negative breast cancer, the only histone family protein to display lactylation was histone H4 K12 lac. The lactylation alteration of histone H4 K12 was greater in cancerous tissues than in nearby controls. These findings indicate that lactylated proteins may be useful in predicting the prognosis of patients with tumors [135]. Chemotherapy resistance, distant metastasis, and local recurrence are characteristics of breast cancer. Consequently, it is imperative to identify more potent treatment targets to increase the overall survival of BC patients. Gui’s study established that lactylation levels in TNBC tissues were greater than those in normal tissues and that elevated lactylation levels within the nucleus could be indicative of RFS in patients with TNBC. GSEA and hub gene screening suggested that nuclear lactylation likely plays an oncogenic role in TNBC via ribosomal subunit synthesis/assembly and aminoacyl‑tRNA production pathways [136]. Deng et al. established a Cox model involving CCR7, IGFBP6, NDUFAF6, OVOL1 and SDC1, and the risk score could be used as a separate biomarker for BC. Moreover, Kla was found to be significantly associated with the BC immune microenvironment, drug therapy, and immunotherapy, and it was also linked to the activation of multiple KEGG pathways related to BC. These findings suggest that Kla is a potential future therapeutic target for BC [137]. Moreover, Jiao et al. used seven prognostic genes (RAD51, NEK10, PCP2, IDO1, CASP14, CLSTN2, and IGHG1) to calculate a prognosis score, which correctly forecasted the outcomes of patients. These findings provide insights into the tumor immune microenvironment [138]. Research into lactylation in breast cancer is still nascent, with substantial scope for further exploration. While current studies primarily focus on histone lactylation, the investigation into non-histone proteins remains underexplored and could reveal significant insights into tumor biology and potential therapeutic targets.

## Novel Kla-based anticancer strategies

The processes of lactate production and transport and lactylation activities and their effector proteins can all be targeted [139–143]. The glucose concentration and rates of glycolysis impact Kla levels, indicating that they are dependent on lactate levels. Therefore, tumor progression can be inhibited by reducing the intracellular lactate concentration or inhibiting lactate activity. The diagnostic and therapeutic goals related to Kla can be classified into two categories: therapies aimed at Kla-regulatory enzymes or the alteration of Kla itself. Notably, greater advancements have been made in the former because of the relative ease of manipulating a single protein rather than a single amino acid. We next discuss the most recent advancements in targeted therapy research (see Table 2).

**Table 2:** Therapeutic approaches targeting the lactate/lactylation axis

Disease	Target	Inhibitor	Effect	References
Breast cancer	MCT1	AR-C155858	Absorption of lactate	[144]
	MCT1	AZD3965	Absorption of lactate	[145]
	PDHK	Dichloroacetate	Generation of lactate	NCT01029925, NCT01386632, etc
	Hexokinase	2-DG	Generation of lactate	NCT00096707, NCT00588185, etc
	Hexokinase	3-BrPA	Generation of lactate	[146]
	Hexokinase	Tristetraprolin	Generation of lactate	[147]
	Hexokinase	Lonidamine	Generation of lactate	[148]
	GPR81	LRH7-G5	Absorption of lactate	[149]
	HAT	Garcinol	Generation of lactate	[150]
	LDHA/LDHB	Quinoline- 3-sulfonamides	Generation of lactate	[151]
Neuroblastoma	MCT1	SR13800	Absorption of lactate	[152]
Colorectal cancer	MCT1	AR-C155858	Absorption of lactate	[144]
	HAT	Garcinol	Generation of lactate	[150]
Pancreatic cancer	MCT1	7 ACC2	Absorption of lactate	[153]
Myeloma	MCT4	Syrosingopine	Excretion of lactate	[154]
Liver cancer	LDHA	GSK2837808 A	Generation of lactate	[155]
	LDHA/LDHB	Quinoline- 3-sulfonamides	Generation of lactate	[151]
	Hexokinase	3-BrPA	Generation of lactate	[146]
Prostate cancer	LDHA	Oxamate	Generation of lactate	[156]
	Hexokinase	2-DG	Generation of lactate	NCT00096707,NCT00588185,etc
	GPR81	Curcumin	Absorption of lactate	NCT05045443,NCT04731844,etc
Lung cancer	LDHA	Oxamate	Generation of lactate	[156]
	LDHA	Galloflavin	Generation of lactate	[157]
	PDHK	Dichloroacetate	Generation of lactate	NCT01029925,NCT01386632,etc
	Hexokinase	2-DG	Generation of lactate	NCT00096707,NCT00588185,etc
	Hexokinase	Lonidamine	Generation of lactate	[148]
	HAT	Garcinol	Generation of lactate	[150]
	GCN5	CPTH6	Generation of lactate	[158]
	MCT1	VB124	Absorption of lactate	[159]
Bladder cancer	Hexokinase	3-BrPA	Generation of lactate	[146]
Melanoma	Hexokinase	Lonidamine	Generation of lactate	[148]
	p300/CBP	A- 485	Generation of lactate	[160]
Nasopharynx cancer	HDAC	ITSA- 1	Generation of lactate	[161]
Leukemia	GCN5	CPTH6	Generation of lactate	[158]
	MCT1	AZD3965	Absorption of lactate	NCT01791595
	MCT1	SR13800	Absorption of lactate	[152]
	GPR81	Curcumin	Absorption of lactate	NCT05045443, NCT04731844, etc
Pituitary adenoma	p300/CBP	A- 485	Generation of lactate	[160]

### Targeting the production or transport of lactate

**Fig. 4:**
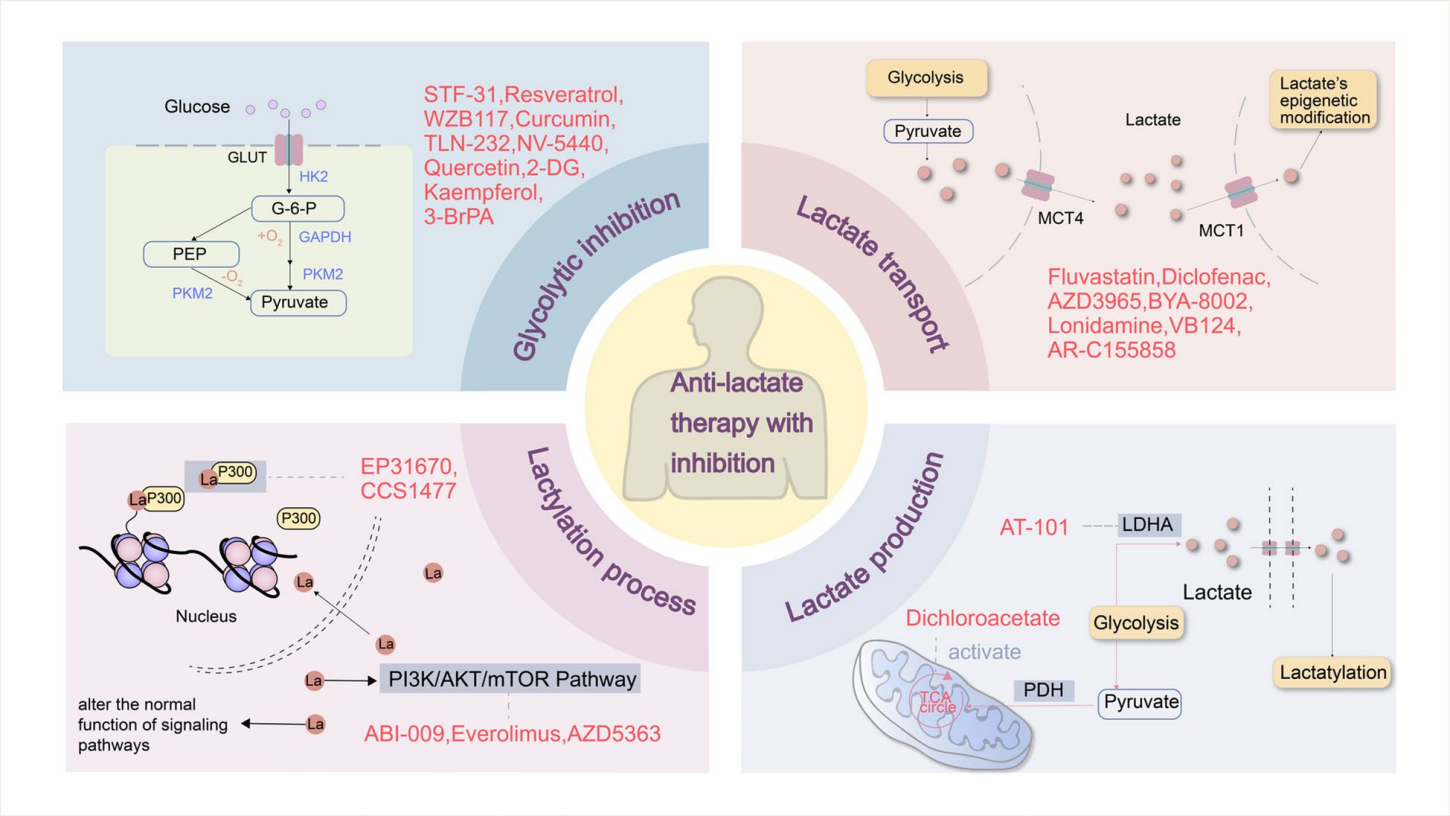
Anti-lactate production and lactylation inhibition strategies. Glucose enters cells via GLUT transporters, where it is processed by enzymes such as PKM and HK2 to produce pyruvate. By blocking these enzymes, lactate generation can be decreased. Pyruvate can enter mitochondria to produce acetyl-CoA by PDH for involvement in the TCA cycle, or it can be converted to lactate by LDH [67, 163, 164]. Lowering lactate generation can be achieved by activating PDH or inhibiting LDH. Lactate inflow and outflow from cells are stopped by blocking MCT1 and MCT4 [145, 174, 175]. Lactate function can be suppressed by interfering with lactate-related signaling pathways, such as the PI3 K/AKT/mTOR pathway. The epigenetic modification of lactate is inhibited through the suppression of lactylation activities caused by the inhibition of p300 [176]

During glycolysis, LDH is a crucial enzyme in the conversion of pyruvate to lactate. Baumann et al. reported a considerable reduction in the carcinogenicity of tumor cells deficient in LDH; additionally, the metabolic profile of the cells was disrupted, and their ability to proliferate was greatly reduced following LDH knockdown [162]. According to studies on lactylation, lactylation is inhibited and the subsequent lactylation pathways are blocked when LDH inhibitors (such as sodium oxamate) are used to downregulate lactate metabolism [14, 67] (Fig. 4). There are now several proven LDH inhibitors, some of which are in phase I and II clinical trials [67, 163, 164]. Three heterogeneous metabolic pathway-based subgroups (MPSs) with unique metabolic characteristics were identified from TNBC samples. The glycolytic subtype MPS2 has elevated nucleotide and carbohydrate metabolism. According to Shao et al., anti-LDH therapy may increase the susceptibility of MPS2 TNBCs to immune checkpoint inhibitors [165]. Galloflavin, a derivative of gallic acid, is a significant antiglycolytic drug that inhibits both LDHA and LDHB in TNBC and estrogen receptor-positive/progesterone receptor-positive (ER +/PR +) breast cancer [166]. Oxamate, another LDHA inhibitor, has been investigated in the TNBC and ER +/PR + breast cancer. Another strong LDHA inhibitor that has been thoroughly investigated in TNBC and ER +/PR + breast cancer is N-hydroxy indole (NHI), and FX- 11, a gossypol analog that targets LDHA, has been thoroughly investigated in ER +/PR + breast cancer [8, 9]. In a trial at National Cancer Institute, referred to as NCI- 006, a new LDH inhibitor has shown efficacy in preclinical models [167]. The lack of strong *in vivo* anticancer effects of the majority of LDH inhibitors, however, may be caused by issues with their metabolic stability, inhibitory efficacy, or mode of delivery. Therefore, more studies targeting LDH inhibitors need to be conducted. Another effective approach has been to suppress the expression of genes upstream of LDH, such as hypoxia-inducible factor [168], the oncogene MYC (encoding c-Myc) [169], heat-shock factor 1 [40], cAMP response element-binding protein (CREB) [170], and MCTs. MCTs, which have four isoforms, MCT1 - 4, catalyze the proton-chain transport of several monocarboxylates, including lactate, across the plasma membrane. MCT1 and MCT4, each with distinct substrates and inhibitor affinities, are known to be overexpressed in a variety of cancers [171], such as breast [51], liver [172], and bladder cancers [173]. Research has demonstrated the efficacy of AZD3965, a dual MCT1 and MCT2 inhibitor, in the management of breast cancer [145]. A phase I clinical trial for advanced solid tumors and lymphomas, including breast cancer (NCT01791595), is currently being conducted in the UK to examine AZD3965. As reported by Guan, AR-C155858 may be an MCT1 substrate, and ARC155858 and AZD3965 both exhibit gradual reversible suppression of MCT1-mediated L-lactate uptake in breast cancer cells [10]. Preclinical research suggests that lactate release into the TME is reduced and antitumor immunity is increased when anti-PD- 1 therapy is combined with the MCT1 inhibitor AZD3965 [11].

### Targeting lactylation enzymes and specific lysine lactylation

In recent decades, interest in applying “writers, readers, erasers” to target histones or the epigenetic regulation of DNA as a cancer therapy has increased. Zhang et al. demonstrated that P300, the first lactylase to be identified, mediates lactylation [14]. The only CBP/P300 inhibitor presently undergoing phase IB/IIA clinical studies is Cell Centric’s CCS1477, a potentially effective treatment for advanced drug-resistant prostate cancer and hematologic malignancies [176] (Fig. 4). The identification of sirtuins and class I histone HDAC1 - 3 as *in vitro* delactylases was originally reported by Zhao et al. in 2022 [88]. HDAC1 - 3 inhibitors are a class of tumor therapeutic targets that have been studied for 30 years, tested in clinical trials, and demonstrated efficacy against a variety of malignancies [177, 178]. However, these findings were based on acetylation evaluations. Lactylation may have the same or stronger effects as acetylation, given the similarities in writers, erasers, and metabolic processes between the two. As there is a direct relationship between Kla and tumor formation, targeting lysine Kla in cancer cells is plausible. Wang et al. discovered that nonhistone proteins might also function as SIRT3 substrates; they also showed that SIRT3 might prevent the progression of HCC cells by blocking the K348 lactylation of the cell cycle protein CCNE2 [179]. This finding suggests that the use of a lactylation inhibitor could be a novel way to prevent HCC progression. According to previous reports, oxamate reduces histone H3 K18 lactylation, which in turn reduces lactate synthesis and decreases the activities of the CD39, CD73, and CCR8 gene promotors. Pan et al. discovered a triterpene anticancer medication that interferes with H3 K9 la and H3 K56 la Kla to prevent liver cancer stem cells from proliferating [180]. Since H3 Kla is a common tumor site associated with Kla, preventing tumor development by reducing H3 Kla appears to be a realistic targeted treatment strategy. By focusing on PKM2, Fargesin suppressed H3 histone lactylation and aerobic glycolytic signaling pathways in A549 NSCLC cells [181].

### Adjuncts to clinical therapy

Because of their safety and specificity, single-site precision interventions offer promising avenues for future studies. However, it is still difficult to create treatments that specifically target lactylation changes and associated processes. Therefore, examining how current treatments interact with lactylation changes is a more clinically viable strategy. The goal is to optimize current medications and increase their antitumor efficacy by reducing the negative effects of lactylation changes in cancer treatments. When immunotherapy is matched to particular molecular characteristics of tumors, the best outcomes can be obtained. Therefore, altering lactylation in the tumor microenvironment may improve the effectiveness of immunotherapy. The production of Treg cells is mechanistically modulated by lactate via the lactylation of Lys72 in MOESIN, which can promote tumor cell immune escape. Thus, increasing immunotherapy effectiveness by limiting MOESIN Kla is possible. When anti-PD- 1 therapy and lactate dehydrogenase inhibitors are combined in animal models, tumor sizes are noticeably smaller than when PD- 1 therapy is used alone [155]. By affecting immunosuppressive immune cells, including regulatory and myeloid-derived suppressor T cells in the TME, lactylation modifications may have beneficial antitumor effects. In individuals who do not respond to immunotherapy, medicines that target lactylation changes may help reverse the immunosuppressive “cold” tumor microenvironment, increasing immunotherapy response rates through individualized treatment plans. Homologous recombination repair is a process for reversing DNA damage. Recent research has indicated that the enzyme MRE11, which governs homologous recombination repair, undergoes significant lactylation facilitated by the p300 enzyme. Because highly lactylated MRE11 has a relatively high affinity for binding DNA, it promotes homologous DNA recombination and results in chemotherapy resistance. The effectiveness of platinum-based or PARP inhibitor chemotherapeutic medications against tumors has been shown to considerably increase after the use of small-molecule peptides that target the lactylation of MRE11 [127]. SMC4 promotes a diapause-like transition in colorectal cancer by upregulating the expression of glycolytic enzymes, which increases lactate levels and lactylation changes inside tumors. By further promoting the expression of ATP-binding cassette transporter (ABC) transporters, these alterations decrease the sensitivity of tumors to chemotherapy [182]. Furthermore, Tan reported that cisplatin resistance in bladder cancer is promoted by the H3 K18 la-driven major transcription factors YBX1 and YY1 [183]. NBS1 lactylation driven by lactate facilitates homologous recombination (HR)-mediated DNA repair. Moreover, a reduction in lactate is achieved through the genetic depletion of lactate dehydrogenase A (LDHA) or stiripentol, an inhibitor of lactate dehydrogenase A used in clinical settings to treat epilepsy, which inhibits NBS1 K388 lactylation, reduces the effectiveness of DNA repair, and overcomes chemotherapy resistance [82]. These findings suggest that treating cancers with a combination of chemotherapy and Kla inhibition may exert therapeutic efficacy. One important targeted therapy modality is antiangiogenic therapy [184–186]. Metastatic colorectal cancer is significantly impacted by the antiangiogenic treatment medication bevacizumab. Nevertheless, the overall effectiveness of bevacizumab is limited by individual resistance. High levels of histone H3 lysine 18 lactylation (H3 K18 la) in colorectal cancer cells are caused by aerobic glycolysis, which stimulates the transcription of RUBCNL; this promotes autophagy by facilitating the maturation of autophagosomes, and it also plays a role in the carcinogenesis and spread of colorectal cancer. Combining autophagy inhibitors with histone lactylation inhibitors has been shown in animal trials to increase the effectiveness of bevacizumab therapy [105]. By reducing intracellular lactate synthesis, evodiamine, a naturally occurring antitumor chemical, prevents the lactylation of hypoxia inducible factor 1 subunit alpha (HIF1a), hence diminishing the inhibitory effect of HIF1a on the antiangiogenic drug Sema3 A [187]. Despite the differences between breast cancer and other cancers, we can still learn from this approach.

The majority of lactylation-targeting strategies still rely on the suppression of lactate production, transport, signal transduction, and even glycolysis. Glycolysis-related mechanisms for metabolism-regulating medications include a range of biological processes. It is still very difficult to obtain effective results in tumor cells with very little disturbance to regular cell metabolism. However, the main goal to effectively target lactylation in particular and offer new targets for tumor therapy is to continue researching and identifying the “writers,” “erasers,” and “readers” of lactylation modification. Moreover, since lactylation frequently occurs in regular physiological functions, it is crucial that medications that target lactylation target cancer cells specifically while avoiding unnecessary damage to healthy organs. Notably, directly targeting lactylation-modifying enzymes requires a careful evaluation of potential consequences for normal intracellular acetylation levels because these enzymes frequently play roles.

## Concluding remarks and future perspectives

Lactate and protein lactylation play important roles in the breast and may have additional functions since they are involved in energy metabolism and signaling in the breast in both healthy and diseased circumstances. Many studies have demonstrated the critical role of lactate in remodeling the TME, controlling tumor cell metabolic reprogramming, and controlling antitumor immunity. Lactate modification is a critical process. Metabolic reprogramming increases the production of lactate in breast cancer, after which lactate accumulates in the tumor microenvironment, leading to an acidic TME, which promotes tumor growth, angiogenesis, metastasis, treatment resistance, and immunosuppression. Moreover, lactate induces lactylation, a recently identified epigenetic alteration that has been observed to be elevated in many tumors. Histone Kla has the ability to directly dock epigenetically and promote the production of oncogenes; moreover, the lactylation of nonhistone proteins has emerged as a crucial regulatory mechanism in numerous tumors. Owing to the ability of immune cells to penetrate tumor tissues, Kla may also promote tumor immunosuppression. Kla may become a significant avenue for tumor-targeted medicine. Consequently, additional research on Kla in tumors may help establish crucial links between metabolism and the epigenetics of malignancies. These findings may also pave the way for targeted treatments based on the enzymes or oncogenic pathways required for Kla.

## Data Availability

No datasets were generated or analysed during the current study.
